# Pupal remodeling and the evolution and development of alternative male morphologies in horned beetles

**DOI:** 10.1186/1471-2148-7-151

**Published:** 2007-08-29

**Authors:** Armin P Moczek

**Affiliations:** 1 Department of Biology, Indiana University, Bloomington IN, USA

## Abstract

**Background:**

How novel morphological traits originate and diversify represents a major frontier in evolutionary biology. Horned beetles are emerging as an increasingly popular model system to explore the genetic, developmental, and ecological mechanisms, as well as the interplay between them, in the genesis of novelty and diversity. The horns of beetles originate during a rapid growth phase during the prepupal stage of larval development. Differential growth during this period is either implicitly or explicitly assumed to be the sole mechanism underlying differences in horn expression within and between species. Here I focus on male horn dimorphisms, a phenomenon at the center of many studies in behavioral ecology and evolutionary development, and quantify the relative contributions of a previously ignored developmental process, pupal remodeling, to the expression of male dimorphism in three horned beetle species.

**Results:**

Prepupal growth is not the only determinant of differences in male horn expression. Instead, following their initial prepupal growth phase, beetles may be extensively remodeled during the subsequent pupal stage in a sex and size-dependent manner. Specifically, male dimorphism in the three *Onthophagus *species studied here was shaped not at all, partly or entirely by such pupal remodeling rather than differential growth, suggesting that pupal remodeling is phylogenetically widespread, evolutionarily labile, and developmentally flexible.

**Conclusion:**

This study is the first to document that male dimorphism in horned beetles is the product of two developmentaly dissociated processes: prepupal growth and pupal remodeling. More generally, adult morphology alone appears to provide few clues, if any, as to the relative contributions of both processes to the expression of alternative male morphs, underscoring the importance of developmental studies in efforts aimed at understanding the evolution of adult diversity patterns.

## Background

Phenotypic diversity is produced by changes in ontogenetic processes occurring at earlier developmental stages. Such changes may be brought about by allelic differences among individuals, differences in ontogenetic environment, or as is probably the case for the majority of phenotypic traits, both. Evolutionary biologists are accustomed to deducing ontogenetic properties based on ontogenetic outputs rather than by studying development as a process. For example, the degree of fluctuating asymmetry between paired adult structures has been assumed by many studies to be indicative of degree of developmental instability and integration [1-3], even though few studies made the effort to investigate the developmental underpinnings of asymmetry [4-6]. Comparative studies of scaling relationships between adult body parts continue to be used heavily to explore the ontogenetic basis of allometries and the evolution of shape [7-11], yet actual attempts to understand formation and growth of said parts during ontogeny are rare or absent. Similarly, considerable literature investigates the developmental costs of secondary sexual trait expression based primarily or solely on examination of adult individuals [12-15]. Here I explore the ontogenetic basis of male horn polyphenism in three closely related species of horn-dimorphic beetles and illustrate how relatively simple developmental studies can be sufficient to advance a deeper understanding of how ontogeny mediates phenotypic evolution and diversification.

### Diversity and biology of Onthophagus beetles

*Onthophagus *beetles have become a popular study system for exploring the interplay between development and evolution during phenotypic diversification [reviewed in [16,17]]. Horn expression in *Onthophagus *beetles varies between species, populations, sexes, and alternative morphs within sexes, with varying contributions of environmental and genetic factors on each of these levels. Species differ heritably in number, location, and shape of horns [18-20]. Genetic differences also determine sexual dimorphisms within the majority of species [13,21]. Typically, only males express fully developed horns while females express no or greatly reduced horns [19]. Phenotypic diversity can be similarly extreme among males within the same population, though here environmental determination predominates. Only males that as larvae have access to optimal feeding conditions eclose to large body sizes and develop a full set of horns, while male larvae with access to sub-optimal feeding conditions eclose to a smaller adult size and remain more female-like and largely hornless [22-25]. The exact scaling relationship, or allometry, between male horn length and body size can differ dramatically between species in shape, slope, and amplitude, and reflects evolved differences in scaling. Consequently, phenotypic differences between large, horned or "major" males and their smaller, hornless or "minor" male counterparts may be more extreme in some species than others. Lastly, scaling relationships may also diverge within species where they can be affected by differences in population-wide environmental conditions [26,27] or reflect genetic divergence in allopatry [28-30]. The present study focuses on the development and evolution of (i) thoracic and head horns, (ii) male dimorphisms in thoracic and head horn expression, and (iii) interspecific differences in degree and kind of male dimorphism in three *Onthophagus *species with a conspicuous male dimorphism.

### Developmental basis of beetle horns

Beetle horns originate as epidermal outgrowths late in larval development during the prepupal stage [31]. At the onset of the prepupal stage the larval epidermis lining the larval cuticle detaches from the cuticle. Selected epidermal regions then undergo rapid cell proliferation, causing epidermal tissue to become compacted and often folded underneath the larval cuticle. Upon shedding the old larval cuticle the animal is then able to assume its pupal shape, including expansion of pupal horn primordia, followed by hardening of the pupal cuticle over the next several hours. Prepupal horn growth is a dynamic and rapid process and in some species 48 hours are sufficient to transition from initial prepupal apolysis to pupal ecdysis [31]. These dramatic shape changes not withstanding, the development of beetle horns is in fact similar to that of traditional appendages, such as legs, mouthparts, or wings, in most holometabolous insects [31-34], with the only exceptions occurring in the higher flies, the wings of butterflies, and some beetles [reviewed in [35,36]]. Differences in degree of adult horn expression between or within species has been taken implicitly or explicitly as evidence of differential growth of these structures [8,10-14,16,37-43]. Recent studies contradict this notion and suggest that explosive growth in the prepupa may, at least in some species, be followed by extensive remodeling of morphology during the pupal stage, in some cases permitting the complete loss of horns and the metamorphosis of a fully horned pupa into an entirely hornless adult [17,21,31,44,45]. Here I investigate pupal remodeling of horn expression in three *Onthophagus *species that have been the subject of many previous studies because of their pronounced male dimorphisms and quantify the contribution of pupal remodeling relative to differential prepupal growth in the development and evolution of intra- and interspecific diversity.

## Results

**Figure 2:**
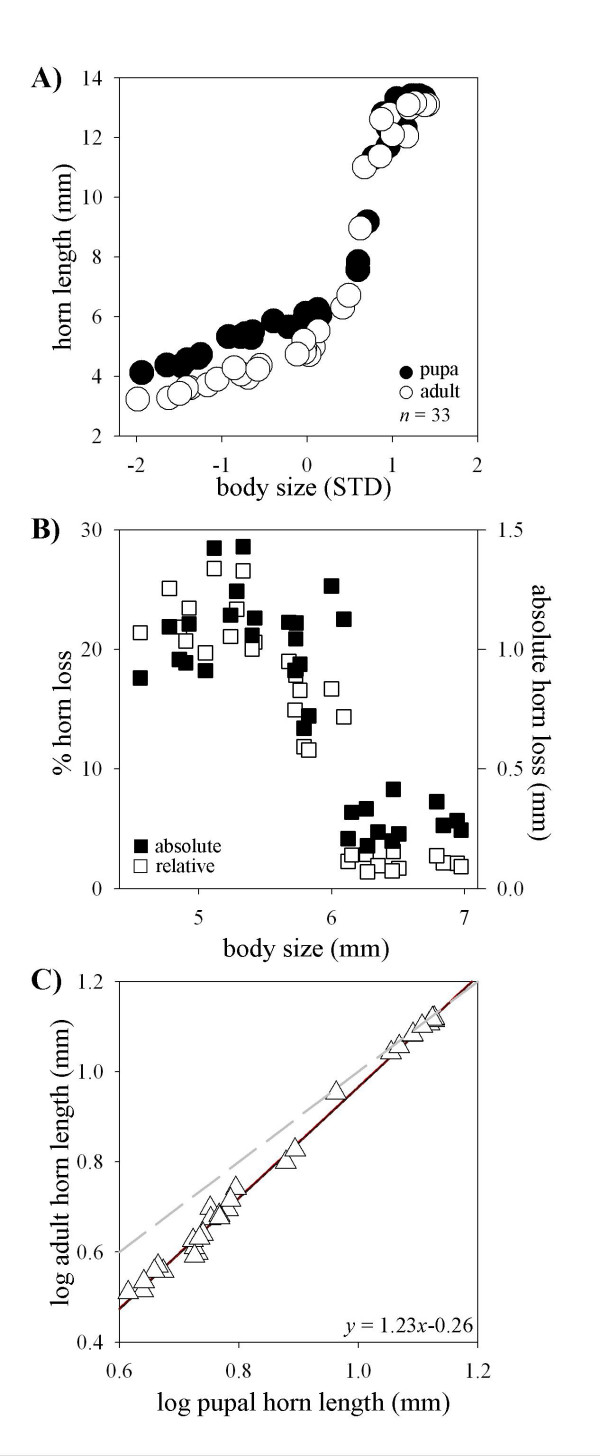
Ontogenetic changes in allometric scaling between body size and thoracic horn length in male *Onthophagus nigriventris*. **(A) **Scaling relationship between body size (presented as standard deviations away from mean) and horn length in male pupae (●) and corresponding adults (○). Allometries differ significantly both in amplitude and slope. **(B) **Absolute (■ right) and relative (□ left) loss of pupal horn length as a function of adult male body size. Both absolute and relative horn loss decline drastically with adult size.**(C) **Log-log plot of pupal against adult horn length. Gray line indicates expectation if adult horn length is a direct reflection of pupal horn length (y-intercept = 0, slope = 1). Regression analysis shows that the y-intercept is significantly different from 0 (indicating pupal remodeling) and the slope is significantly greater than 1 (indicating that remodeling occurs to a greater degree in minor compared to major male morphs). Red lines indicate 99% confidence intervals.

In *O. nigriventris*, scaling relationships between body size and horn length changed significantly from the pupal to the adult stage (Fig. 2A). Specifically, both amplitude and steepness of the slope increased significantly from the pupal to the adult stage, creating a greater and more sudden disparity between minor and major male morphs (amplitude: T_64 _= 2.76, *p *= 0.0075; slope: T_64 _= 2.53, *p *= 0.0139). Both relative and absolute loss of horn length decreased quickly with male size (Fig. 2B, F_rel _= 127; *p *< 0.0001; F_abs _= 42; *p *< 0.0001). Small males commonly lost >1 mm (> 20%) of horn length compared to ~0.25 mm (< 5%) in large males. Log-log plots of pupal against adult horn length showed that hornless, minor males fell below the line expected if adult horn length was a direct, unaltered reflection of pupal horn length (indicated by gray line in Fig. 2C), whereas horned, major males appeared right on that line. This observation was backed up by regression analysis, which yielded a negative y-intercept significantly different from 0 (T_32 _= 18.95, *p *< 0.0001), confirming that horn lengths of at least some males decreased from the pupal to the adult stage, and with a slope highly significantly greater than 1 (Fig. 2C; T_32 _= 14.63; *p *< 0.0001), supporting that this decrease was strongest for small males.

**Figure 3:**
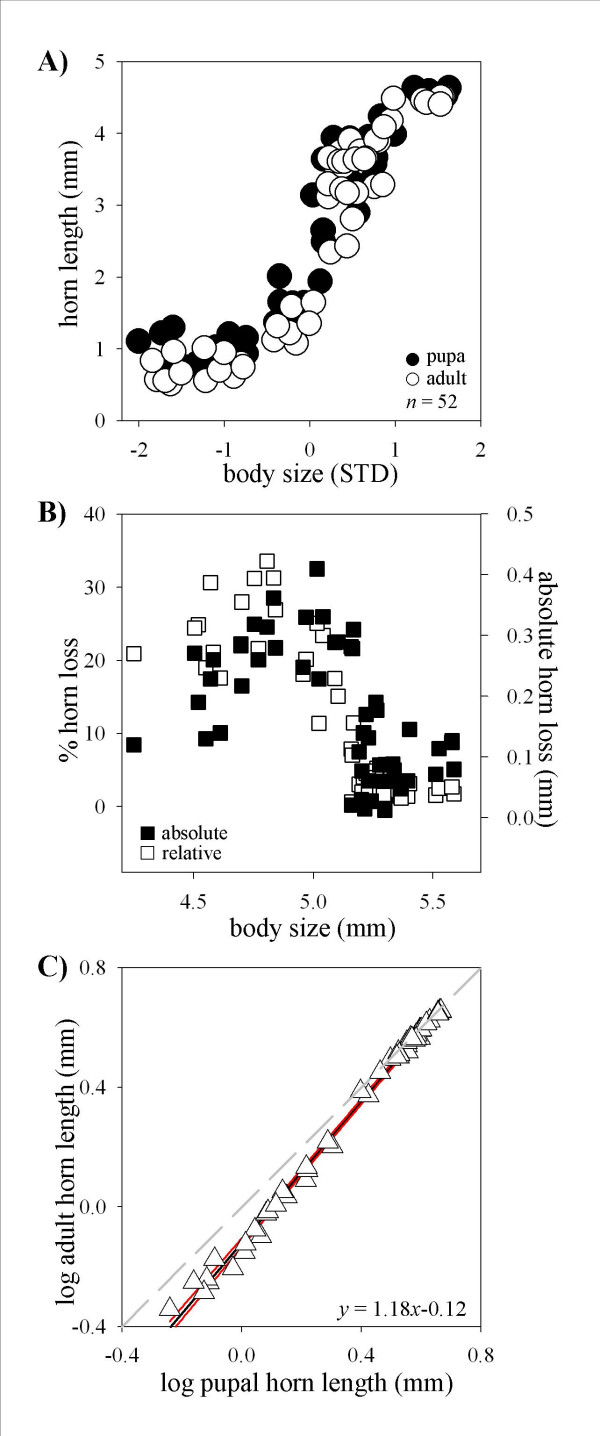
Ontogenetic changes in allometric scaling between body size and head horn length in male *Onthophagus taurus*. **(A) **Scaling relationship between body size (presented as standard deviations away from mean) and horn length in male pupae (●) and corresponding adults (○). There are no significant differences between pupal and adult allometries. **(B) **Absolute (■ right) and relative (□ left) loss of pupal horn length as a function of adult male body size. Relative horn loss declines steadily with adult size, whereas absolute horn loss first increases and reaches a maximum in medium-sized males before declining again to near zero values in large males. **(C) **Log-log plot of pupal against adult horn length. Gray line indicates expectation if adult horn length is a direct reflection of pupal horn length (y-intercept = 0, slope = 1). Regression analysis shows that the y-intercept is significantly different from 0 (indicating pupal remodeling) and the slope is significantly greater than 1 (indicating that remodeling occurs to a greater degree in minor compared to major male morphs). Red lines indicate 99% confidence intervals.

In *O. taurus*, scaling relationships between body size and horn length did not change significantly from the pupal to the adult stage (Fig. 3A). Neither amplitude nor steepness of the slope increased significantly from the pupal to the adult stage. However, both relative and absolute loss of horn length still showed a significant effect of body size. As with *O. nigriventris*, relative horn loss decreased rapidly with male size (Fig. 3B, F_rel _= 110; *p *< 0.0001), however, absolute horn loss first increased, reaching a peak in medium-sized males (x_0 _= 4.83 ± 0.027 mm; *p *= 0.0001), before declining again to near zero values in large males (F_abs _= 28.37; *p *< 0.0001). As with *O. nigriventris*, log-log plots of pupal against adult horn length yielded a negative y-intercept (T_51 _= 21.98, *p *< 0.0001) and a slope highly significantly greater than 1 (Fig. 3C; T_51 _= 14.96; *p *< 0.0001). Also as before, hornless, minor males fell below the line expected if adult horn length was a direct reflection of pupal horn length whereas horned, major males appeared right on that line.

**Figure 4:**
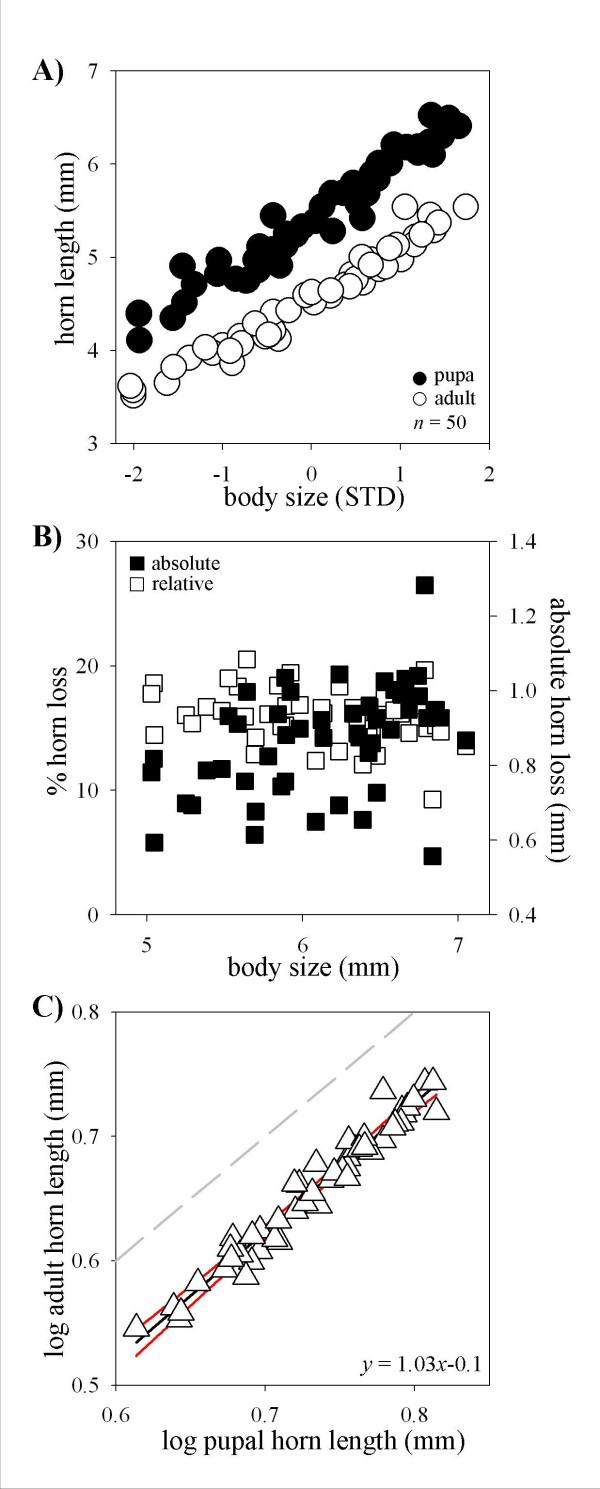
Ontogenetic changes in allometric scaling between body size and thoracic horn length in male *Onthophagus binodis*. **(A) **Scaling relationship between body size (presented as standard deviations away from mean) and horn length in male pupae (●) and corresponding adults (○). Pupal and adult allometries differ significantly in y-intercept but not slope. **(B) **Absolute (■ right) and relative (□ left) loss of pupal horn length as a function of adult male body size. Relative horn loss exhibits a marginally significant negative correlation with adult size, whereas absolute horn loss increases significantly with adult size. **(C) **Log-log plot of pupal against adult horn length. Gray line indicates expectation if adult horn length is a direct reflection of pupal horn length (y-intercept = 0, slope = 1). Regression analysis shows that the y-intercept is significantly different from 0, but that the slope is indistinguishable from 1 (indicating that remodeling occurs similarly for all males regardless of size). Red lines indicate 99% confidence intervals.

**Figure 5:**
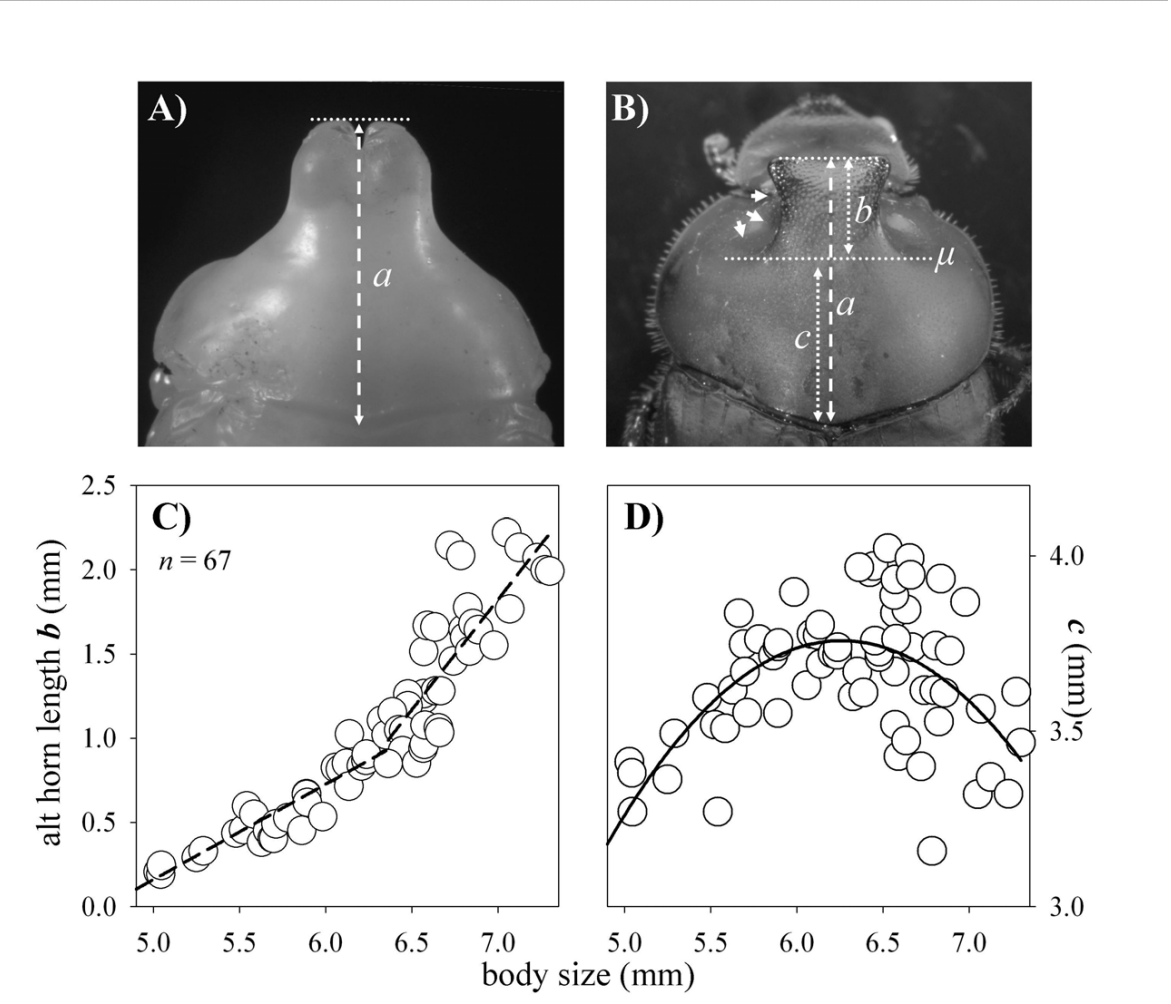
Dorsal view of a male *O. binodis *as **(A) **pupa and **(B) **corresponding adult. Note presence of pronounced lateral concavities, or depressions, in the prothorax of the adult but not pupae (marked by arrows on left side of the adult). *a *indicates pupal and adult horn length measurements used in this study, which failed to reveal a male dimorphism in this species. *b *indicates adult horn length measurement used by previous studies, which relies on *μ *as the posterior landmark, marked by the posterior-most edge of the two lateral prothoracic depressions. **(C) **Use of *μ *as posterior landmark, and *b *as a measure of horn length, recovers a male horn dimorphism in *O. binodis *adults similar to what has been documented in earlier studies. **(D) **Scaling relationship between *c *(which measures the proportion of the prothorax that does not participate in concavity formation) and adult male size. *c *initially increases with male size and then declines rapidly in large males, suggesting that large males devote a disproportionately larger fraction of their lateral prothorax toward exposing the medial thoracic horn (solid line = log normal regression: r^2 ^= 0.42, *p *< 0.0001; in contrast a linear regression (not shown) fails to yield a significant fit; r^2 ^= 0.06; *p *= ns). Combined, these results are consistent with the hypothesis that dimorphic remodeling of the lateral prothorax during the pupal stage, rather than dimorphic growth of actual horn tissue during the prepupal stage, generates male dimorphism in *O. binodis*. To increase statistical power data shown in (C) and (D) include an additional 23 male *O. binodis *reared under identical conditions excpet that no measurements were taken during the pupal stage.

In the third species, *O. binodis*, results were yet again different. The scaling relationships between body size and horn length changed significantly from the pupal to the adult stage, however, unlike the previous two species all adults beetles had much shorter horns then their corresponding pupae regardless of final body size (Fig. 4A). As a consequence, y-intercepts of linear regressions on pupal and adult allometries differed significantly (T_98 _= 31.1, *p *< 0.0001). More importantly, allometric slopes also changed significantly, but in a direction opposite to that observed in the previous two species. Adult *O. binodis *had a slightly but significantly shallower slope than the pupae from which they had eclosed (T_98 _= 2.64, *p *= 0.0098). Loss of horn tissue also showed a pattern different from that observed in *O. nigriventris *or *O. taurus *(Fig. 4B). Specifically, relative horn loss only showed a very moderate, if any, decrease with adult size (F_rel _= 4.8; *p *= 0.033; *ns *after corrections for multiple comparisons). In contrast, absolute horn loss exhibited a moderate, yet significant, increase with adult size (F_abs _= 11.25; *p *= 0.0016). Log-log plots of pupal against adult horn length yielded a negative y-intercept significantly different from 0 (T_49 _= 4.38, *p *< 0.0001), further confirming that horn lengths changed from the pupal to the adult stage, but with a slope indistinguishable from 1 (Fig. 4C; T_49 _= 1.026; *p *= ns), indicating that this change was similar for all males regardless of their size. Combined, these data suggest that pupal remodeling does affect male shape but not male dimorphism in this species. Most strikingly, however, this analysis failed to support the existence of a male dimorphism in horn expression in the first place, even though this species has been the subject of several previous studies into the biology of alternative male morphs [4,5,15]. To help resolve this contradiction I measured adults using the same landmarks used by previous studies, as illustrated in Figure 5 (J. Tomkins, pers. communication). Rather than using the scutellum as a posterior boundary, earlier studies used an imaginary line drawn perpendicular to the body axis at the level of two lateral concavities (*μ *in Fig. 5B), or depressions, that characterize the adult prothorax. No such concavities exist in pupae and this measurement can only be made on adults. Use of this alternative posterior landmark recovered a dimorphic scaling relationship very similar to those published previously (Fig. 5C). This suggested that male dimorphism in *O. binodis *may not be a function of horn growth *per se*, but rather a function of the degree to which lateral aspects of the pupal prothorax retract, or "cave in", around the medial prothorax. To further examine this possibility, I calculated the length of the part of the thorax, *c*, that was unaffected by lateral concavity formation for each individual (Fig. 5b). If lateral concavity formation is independent of body size, i.e. the same proportion of the prothorax participates in concavity formation regardless of final adult size, then *c *should increase isometrically with male size. In contrast, if large, major males have larger horns because the lateral prothorax is further caved-in than in small males, *c *should scale with body size with a slope < 1, and possibly even a negative slope. In support of the latter hypothesis, *c *was found to first increase and reach a peak in medium-sized males size before declining again rapidly in larger males (Fig. 5D; log normal regression: r^2 ^= 0.42, *p *< 0.0001). In contrast, a linear regression of *c *on body size failed to yield a significant fit (r^2 ^= 0.06; *p *= ns). Separating small and large males in the analysis further confirmed this observation. *c *was found to increase significantly with body size in males less than 6.4 mm in thorax width (F = 42.74; *p *< 0.0001), but to decrease significantly with body size in males larger than that (F = 12.72; *p *< 0.001). This supports the hypothesis that male dimorphism found in *O. binodis *is not the product of dimorphic growth of horn tissue but instead results from body size-dependent caving-in, or retracting, of the lateral prothorax, exposing a relatively larger thoracic projection in large males compared to their smaller male counterparts.

## Discussion

*Onthophagus *beetles have emerged as a promising model system for exploring the interplay between genetic, developmental, and ecological factors in the genesis of phenotypic diversity [16,17,21,46]. Part of their attractiveness stems from the enormous morphological diversity in secondary sexual traits found between species, sexes, and alternative morphs within sexes, providing opportunities to explore, within a very narrow phylogenetic distance, topics such as the costs and limits of trait elaboration [8,23], developmental and life-history tradeoffs related to secondary sexual trait expression [10-12,43], or the frequency of independent evolutionary events necessary to explain extant patterns of diversity [13,14]. Importantly, the majority of earlier studies implicitly or explicitly assumed that the expression of secondary sexual traits in adult beetles is the consequence of differential activation of growth of these structures during immature development [8,10-12,16,37-40,42,43]. Here I present results on the developmental origin of alternative male dimorphism in three *Onthophagus *species that may help refine this assumption.

In two of the three species studied here, *O. nigriventris *and *O. taurus*, pupal remodeling of horn length occurred in a size dependent manner. Smaller and medium sized-males lost both relatively and absolutely greater amounts of horn tissue compared to their larger male counterparts, causing males to change pupal to adult proportions in a size dependent manner. In large males of both species, pupal proportions were largely maintained in the eclosing adults, whereas smaller and medium-sized males were more extensively remodeled. In *O. nigriventris*, size-dependent pupal remodeling of male horn length significantly altered the scaling relationship between horn length and body size, causing adult allometries to exhibit greater disparity between alternative male morphs and to transition from one morph to the other over a narrower range of body sizes compared to the pupae from which they eclosed. Allometric differences in *O. taurus *were in the same direction as in *O. nigriventris *but were not significant. Overall, however, loss of pupal horn tissue, and remodeling of pupal proportions prior to adult eclosion, were considerably less severe than what has been previously documented for female *Onthophagus *and the development of sexual dimorphisms, where in many cases fully horned pupae have been shown to molt into entirely hornless adults lacking any indication of the earlier existence of a horn [21,44,45]. These results suggest that pupal remodeling can play a moderate, but not dominant, role in the development of some male dimorphisms. Specifically, in species in which prepupal horn growth is already dimorphic, pupal remodeling appears to have the capacity to further exaggerate male dimorphism. Furthermore, given that the relative loss of horn tissue was highest in small to medium-sized males, pupal remodeling in these species also has the potential to further reduce the production of intermediate morphologies from the population of phenotypes generated by dimorphic growth alone.

Results in the third species, *O. binodis*, however, suggest an additional dimension to the role of pupal remodeling. Initial results suggested that pupal remodeling of horn length also occurs in this species, causing pupal proportions to change as pupae become adults, but without a strong dependence on adult size. Instead, pupal horn length changed similarly in all males regardless of final size. Most importantly, however, horn length-body size measurements failed to reveal an actual male dimorphism in both pupae and adults, even though this species has been studied in detail for its male dimorphism by previous studies [10,13,37,38,43], and visual inspection of specimens leaves the strong impression of a significant discontinuity in horn expression when comparing males of different body sizes. Repeat measurements of the same individuals using the same posterior landmark used in earlier studies recovers a male dimorphism similar to what has been previously documented [43]. This alternative, original landmark is formed by two lateral concavities, or depressions of the prothorax, and an imaginary line that can be drawn perpendicular to the body axis at the level of the posterior-most extent of these depressions (Fig. 5), whereas the landmark used initially in this study was formed by the posterior border of the horn-bearing segment, the prothorax. The crucial difference between these two measurements lies in the fact that if horn length is measured using the lateral concavities as a landmark, length of horns becomes a function of not only the extent to which the horn extends forward, but also of the degree to which the lateral depression indent the prothorax. Measuring horn length using the posterior edge of the prothorax as a boundary is independent of the presence or absence of lateral depressions. Subsequent analysis showed that large males indeed allocate much larger proportions of their prothorax to the formation of lateral concavities compared to males of smaller body sizes (Fig. 5D). The complete absence of these concavities in pupae suggests that male dimorphism in *O. binodis *is not the product of dimorphic prepupal growth of horn tissue but instead may result entirely from body size-dependent caving-in, or retracting, of the lateral pupal prothorax, which ultimately exposes a much larger thoracic horn in large males compared to their smaller male counterparts. More generally, this suggests that dimorphisms, such as those found in *O. binodis*, do not necessarily require dimorphic growth of the trait of interest, but may be generated partly or entirely by dimorphic remodeling, or *sculpting*, of the surrounding body regions.

### The developmental basis of pupal horn remodeling

Recent studies on pupal remodeling of female horn expression implicate the local activation of programmed cell death as the most likely proximate mechanism underlying pupal remodeling [[21]; T. Kijimoto, J. Andrews, and A. Moczek unpublished). Programmed cell death (PCD) is a basic physiological process used by all metazoan organisms to remove superfluous or harmful cells and to sculpt organs and body parts during morphogenesis. PCD relies on a tier of phylogenetically highly conserved genetic and developmental processes [47-49], and is executed via apoptosis or autophagy, two processes that differ primarily in the mechanisms used for degradation of the dying cell [50]. In holometabolous insects, PCD-mediated sculpting of pupal body parts is essential to attain their final adult shape and function [51,52]. In extreme cases PCD can even mediate the whole sale loss of entire structures, such as caste-specific degeneration of prepupal wing discs in *Pheidole *ants [53] or sex-specific apoptotic wing degeneration during pupal development of some Lepidoptera [54,55]. In *Onthophagus*, PCD-mediated loss of horn tissue has been implicated in the production of sexual dimorphism via sex-specific removal of entire pupal horns in a number of species [21] and PCD-mediated removal of horn tissue is also a likely mechanism underlying the more subtle pupal remodeling of horn length reported here for *O. nigriventris *and *O. taurus*. Pupal remodeling in *O. binodis*, however, is likely to require additional mechanisms beyond the simple removal of horn tissue through PCD. Specifically, it is difficult to envision how the lateral concavity formation observed in *O. binodis *can be achieved through the simple removal of epidermal cells, as the surface area generated by these concavities appears similarly large, if not larger than the corresponding, initial surface area in the pupal stage. Rather than requiring the removal of tissue, the formation of concavities is likely to require local changes in the orientation and shape of epidermal cells to generate epidermal infolding, similar to what has been documented for the early formation of joints in *Drosophila *appendages [35]. If correct, this would suggests that the formation of lateral concavities in *O. binodis *does not actually free up resources that could be allocated elsewhere, as would be the case with pupal remodeling mediated purely by PCD, but instead would require additional resources to alter shape and orientation of cells. Studies are now under way documenting and quantifying changes in cell shape and orientation during the pupal remodeling stage of horn development. However, independent of exactly which developmental processes mediate pupal remodeling, it already appears clear that they must be able to do so in a sex-, size, and location-specific manner. Preliminary observations implicate juvenile hormone (JH), Insulin (IN), and Epidermal Growth Factor Receptor (EGFR) signaling in the regulation of degree and location of PCD and cell shape changes in pupal horns (T. Kijimoto, J. Andrews, A. Moczek, unpublished). All three pathways are well known for their important roles in the regulation of organ and body size in a wide range of organisms [42,56,57] and experiments are under way to explore the roles of JH, In, and EGFR-signaling during pupal remodeling through comparative gene expression assays as well as functional analysis.

## Conclusion

The data presented here illustrate that alternative male morphologies, at least among the three *Onthophagus *species studied here, are shaped by a minimum of two developmentally dissociated processes: growth of horn tissue during the prepupal stage, followed by remodeling during the pupal stage. In species in which prepupal horn growth is already dimorphic, pupal remodeling appears to have the capacity to further exaggerate male dimorphism and selectively remove intermediate phenotypes from the distribution of phenotypes generated by prepupal growth alone. In contrast, in species such as *O. binodis *male dimorphism may not require dimorphic prepupal growth and instead may be shaped entirely by dimorphic pupal remodeling. Most importantly, adult morphology alone appears to provide few clues, if any, as to the relative contributions of prepupal growth and pupal remodeling to the expression of alternative male morphs, thus underscoring the importance of developmental studies in efforts aimed at understanding the evolution of adult diversity patterns.

## Methods

### Species choice and husbandry

**Figure 1:**
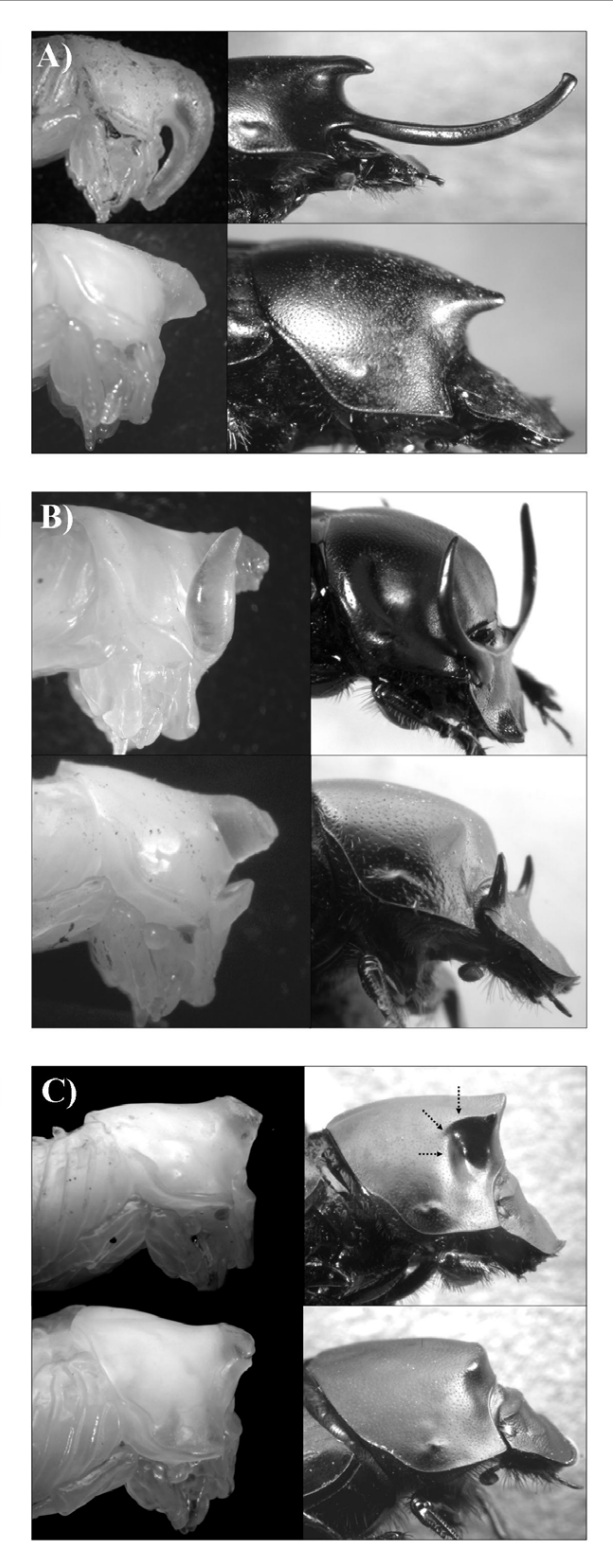
Species used in the present study. **(A) ***Onthophagus nigriventris*, **(B) ***O. taurus*, and **(C) ***O. binodis*. Shown for each species are large horned (major) males (top) and small, hornless (minor) males (bottom) as pupae (left) and corresponding adults (right). Arrows highlight lateral concavity in adult, but not pupal, *O. binodis *referred to in text.

I investigated the relative contribution of pupal remodeling in the development and evolution of sexual dimorphisms by quantifying ontogenetic changes in the allometric scaling between body size and horn length from the pupal to the adult stage and the degree to which ontogenetic changes in allometries differed between males as a function of body size. In particular, I examined the ontogeny of male horn dimorphism in three species (Fig. 1). Large adult male *O. nigriventris *express an enormous thoracic horn, whereas small males express only a small pointy thoracic projection. The transition from small to large horns occurs over a very narrow range of body sizes [58]. *O. binodis *exhibits the same general pattern, though horn size and degree of male dimorphism are considerably reduced compared to *O. nigriventris *[37,38]. Lastly, large male *O. taurus *express two head horns while smaller males remain female-like and largely hornless [59]. Males of all three species use their horns in male fights over access to females, where they function as jousting devices during head-to-head combat and to block entrances to breeding tunnels [38,58,60].

Laboratory colonies of all three species were derived from field populations. *O. taurus *was collected from pastures around Bloomington, IN, and *O. nigriventris *and *O. binodis *were collected from pastures near Waimea, Hawaii. All species were maintained and reared as described previously [44]. Early third instar larvae of each species were transferred from their natural brood ball into 12-well plates to monitor larval development as described previously [61]. First to second-day pupae were measured, weighed, and then returned to their artificial brood ball until adult eclosion. At this stage the pupal epidermis still fully lined the pupal cuticle (Moczek, unpublished) and pupal size measurements are therefore a direct reflection of growth completed prior to the pupal stage. Eclosing adults were retained in brood balls for an additional 3–4 days to allow the adult cuticle to fully harden, then weighed, killed, stored in ethanol, and re-measured as described below.

### Morphometric measurements

Pupae and adults of all three species were measured as described in Moczek [21]. To summarize, pupal and adult thorax width was used as a proxy for body size (for justification see [22,25]. Head horn length in *O. taurus *was measured along the outer edge of one horn beginning at the edge of the eye cavity and ending at the tip of the horn. Thoracic horn length in *O. binodis *and *O. nigriventris *was measured as the distance from the anterior-most point of the prothorax, or "tip" of the horn, to the posterior-most point of the prothorax bordering anteriorly to the scutellum of the second thoracic segment. Measuring horns in this fashion allowed me to unambiguously recognize homologous landmarks in both pupae and adults [21]. For comparison, and to examine possible effects of measurement technique, thoracic horns in *O. binodis *were also measured using a partly different set of landmarks, as in earlier studies [10,13,43]. In these studies *O. binodis *horn length was measured as the linear distance from the tip of the horn to an imaginary line drawn across the prothorax (perpendicular to the body axis) at the level of two lateral prothoracic concavities present in adult males (J. Tomkins, personal communication; indicated in Fig. 1c and 5b). No corresponding landmark exists in pupae and this measurement is therefore restricted to adults. To increase sample size and statistical power in the analysis of adult scaling relationships in *O. binodis *I included measurements of an additional 23 adult males. Males in this additional sample were reared under identical conditions except that no measurements were taken while they were in the pupal stage.

### Statistical analyses

Pupal and adult scaling relationships were contrasted by fitting 4-parameter non-linear regression models (*O. nigriventris *and *O. taurus*) or simple linear regression models (*O. binodi*s) to the data. Pupae are consistenly about 5% larger (in thorax width) than the adults that emerge from them, regardless of sex or species [21]. Pupal and adult scaling relationships were therefore contrasted in two ways, first using untransformed body size measurements, and then again using body size values transformed to standard deviations away from the mean body size. The later comparison was also used to graphically contrast pupal and adult scaling relationships because it centers scaling relationships onto the mean body size characteristic of each developmental stage. I then used T-tests to examine the degree to which possible differences between pupal and adult scaling relationships could be explained by particular regression parameters. Specifically, I examined whether pupal and adult scaling relationships differed in (i) the range of horn expression, or amplitude, which can be interpreted as a measure of the disparity between morphs achievable over a given range of body sizes, and (ii) the steepness of the allometric slope, which can be interpreted as a measure of body size range necessary to achieve the transition from one morph to the other. All analyses were conducted using both untransformed and transformed body size values. To further characterize nature and mechanisms of pupal remodeling I then quantified relative loss of pupal horn length (calculated as the percentage of pupal horn length not retained in the adult) and absolute loss of pupal horn length (calculated as the absolute difference between pupal and adult horn length) for each individual as a function of body size using standard linear regressions. Lastly, to further explore the significance of such changes I regressed log-transformed pupal against adult horn lengths. If adult horn length is a direct reflection of pupal horn length regardless of male size then the slope of such a log-log plot should be 1 with an intercept of 0. If horn length decreases from the pupa to adult by the same magnitude for each individual this should cause the slope to remain 1, but the intercept to become negative. Lastly, if males differ in how they convert pupal to adult horn length as a function of their body size this would be manifest in a change in the slope from 1. I used T-tests to determine whether slopes or intercepts differend significantly from 1 or 0, respectively. I used sequential Bonferroni procedures to correct for multiple comparisons within and between species where this was necessary.

## Authors' contributions

APM is responsible for planning and executing all work involved in this paper.
